# The Story of the Insane from Year to Year

**Published:** 1898-03-12

**Authors:** 


### THE STORY OF THE INSANE FROM YEAR TO YEAR.

Tenth Series.

### WEST RIDING ASYLUM AT WAKEFIELD.

On January 1st, 1896, this asylum contained 1,390 patients, and at the end of the year the numbers had decreased by 7. There were 471 first admissions, 88 re-admissions, 139 recoveries, and 152 deaths. The recoveries stand at 24 80 per cent. on the admissions, and the deaths at 11 02 per cent. on the average number resident. Of the deaths 75 were caused by cerebral and spinal diseases, 16 to consumption, 1 to enteritis, and 1 to enteric fever. Of the year's admissions 92 were stated to owe their insanity to various moral causes, such as domestic trouble, religious excitement, and love. As usual intemperance in drink and hereditary influence were by far the most potent factors, the latter heading the list with 177 and the former accounting for 149. As regards drink as a cause of insanity, Dr. Lewis points out that mere figures cannot be relied on in forming an opinion as to its frequency, and that we should want to know the proportion of drunkards who become insane to those who remain sane, and also the proportion of sober people who become insane to those who remain sane. This is true enough, but as we cannot obtain these data we are at present only justified in saying that drink is a very potent cause of insanity, and that when the breadwinner of a family takes to drink it operates indirectly as a cause of insanity in other members of the family by depriving them of proper food, clothing, and domestic happiness. We are pleased to congratulate Attendant Read, Attendant George Cockram, Nurse Crossland, and Nurse Talbot on having obtained their pensions, and we think every publicity should be given to such grants, as it points out to those who wish to enter asylum service which institutions to apply to. Nursing lectures are given to the staff, and a list of those who have obtained certificates is given at the close of the superintendent's report. We have often expressed favourable opinions concerning the value of these instructions, but we trust the innovation of publishing the names of those of the staff who have obtained certificates will not become common. The committee say that "the progress of the asylum under Dr. Bevan Lewis' able management has been very satisfactory." The weekly cost per head is 9s. 3⅝d.

